# A Novel Association between Two Trypanosome-Specific Factors and the Conserved L5-5S rRNA Complex

**DOI:** 10.1371/journal.pone.0041398

**Published:** 2012-07-31

**Authors:** Martin Ciganda, Kimberly Prohaska, Kristina Hellman, Noreen Williams

**Affiliations:** Department of Microbiology and Immunology and Witebsky Center for Microbial Pathogenesis and Immunology, University at Buffalo, Buffalo, New York, United States of America; University of Texas-Houston Medical School, United States of America

## Abstract

P34 and P37 are two previously identified RNA binding proteins in the flagellate protozoan *Trypanosoma brucei*. RNA interference studies have determined that the proteins are involved in and essential for ribosome biogenesis. The proteins interact with the 5S rRNA with nearly identical binding characteristics. We have shown that this interaction is achieved mainly through the LoopA region of the RNA, but P34 and P37 also protect the L5 binding site located on LoopC. We now provide evidence to show that these factors form a novel pre-ribosomal particle through interactions with both 5S rRNA and the L5 ribosomal protein. Further *in silico* and *in vitro* analysis of *T. brucei* L5 indicates a lower affinity for 5S rRNA than expected, based on other eukaryotic L5 proteins. We hypothesize that P34 and P37 complement L5 and bridge the interaction with 5S rRNA, stabilizing it and aiding in the early steps of ribosome biogenesis.

## Introduction

Ribosomes are complex structures dedicated to translation of mRNAs into protein. Catalysis is performed by the RNA component of the ribosome [1]. The rest of the ribosome (approximately half of its mass) consists of proteins, many of them with unidentified functions [2]. In eukaryotes, ribosome biogenesis takes place in the nucleolus, a specialized subnuclear structure, where transcription of the large RNA precursor is performed by RNA polymerase I, and this transcript undergoes several steps of maturation that involve cleavage at specific sites and assembly with specific proteins (reviewed in [3]). One of the rRNAs, the 5S rRNA, is usually transcribed outside the nucleolus, in the nucleoplasm of the eukaryotic cell (reviewed in [4]). This RNA has a conserved and defined secondary structure. It folds in three arms, consisting of five loops (A through E) and five stems (I through V) [5]. Subsequent binding with the ribosomal protein L5 (eukaryotic homologue of L18p) through its Loop C/Stem III domain [6], leads to the formation of a unique extraribosomal particle [7]. This association is necessary for the stabilization and transport of this rRNA to the nucleolus. L5 is an essential protein both in prokaryotes [8] and in eukaryotes [9].

Our group has recently described the specific interaction of two *Trypanosoma brucei* proteins with 5S rRNA [10], [11]. The parasitic kinetoplastid *T. brucei* belongs to a group of organisms that branched out early in evolutionary history and presents many unique aspects in its molecular biology. They have a large public health impact, causing disease in vulnerable regions of the world and being a major factor impeding development due to enormous economic consequences [12]. In the Americas, *T. cruzi* causes Chagas disease, whereas several subspecies of *T. brucei* are responsible for human African trypanosomiasis and nagana in cattle and domestic animals. Although research on these organisms has accelerated over the last decades, much of their biology remains to be elucidated before they can be tackled effectively. The two 5S rRNA interacting proteins identified in our laboratory, P34 and P37, represent attractive targets in that we have previously shown that they are essential for parasite survival and that they are not present in the host. In fact, they are unique to trypanosomatids.

An association with 5S rRNA has potential implications in the early stages of the process of ribosome biogenesis, namely the stabilization and trafficking of 5S rRNA to the nascent ribosomal particle in the nucleolus. In most well characterized eukaryotes such as *Xenopus laevis,* this process has been extensively described [13] [14], [15] and it is known that only a small number of proteins can bind 5S rRNA through specific protein-RNA contacts, and only the L5 protein forms a pre-ribosomal particle with 5S rRNA. We have shown that the *T. brucei* 5S rRNA establishes an association with P34 and P37 via its Loop A/Stem V domain and that protection by these proteins reaches Loop C at the end of one of its three arms [16]. Since this region partially overlaps with the L5 site of binding [6], an immediate set of question arises: Are P34 and P37 involved in the early stages of ribosome biogenesis? Are they associated with L5? Furthermore, earlier work using RNA interference demonstrated that the absence of P34 and P37 causes a phenotype similar to that caused by the absence of L5 in yeast. Why do trypanosomes need an additional pair of factors to accomplish what other eukaryotes can do with only one? In this paper we address these questions.

## Materials and Methods

### Cell Culture and Extract Fractionation

Procyclic *T. brucei* strain 427 was grown in Cunningham’s media supplemented with 10% fetal bovine serum. Nuclear and subnuclear extracts were prepared for L5/5S studies as described previously [17]. Briefly, 2.5×10^10^ procyclic cells were pelleted and resuspended in 8% polyvinylpyrrolidone (PVP, Sigma), containing 0.05% Triton X-100 (Sigma), 5 mM DTT, mammalian protease inhibitor mixture (Sigma) and solution P (100 mg phenylmethylsulfonyl fluoride, 2 mg pepstatin A in 5 mL of ethanol). Cells were homogenized and passed through a 25 gauge needle. The lysate was underlaid with 0.3 M sucrose in 8% PVP, solution P, 1 mM DTT and protease inhibitor mixture and sedimented at 11,000× g. The pellet containing the crude nuclear extract was resuspended in 8 mL of 2.1 M sucrose in 8% PVP, DTT, protease inhibitor cocktail, and solution P. This mixture was applied to a discontinuous gradient in a SW28 tube (bottom to top: 2.3 M sucrose, 2.1 M sucrose, 2.01 M sucrose). After sedimentation at 100,000× g for 2 hours at 4°C, nuclei were recovered from the 2.1/2.3 interface. Nucleoli were separated by sonication and recovery on a sucrose step gradient (2.50 M sucrose, 2.25 M sucrose and 1.75 M sucrose) at the 2/2.5 M interface. Each subcellular fraction was subjected to western blot analysis using specific marker antibodies to confirm purity. NOG1 and PGK (both gifts from Dr. Marilyn Parsons, SBRI) were used as nucleolar and cytoplasmic markers, respectively. TBP (gift from Dr. Vivian Bellofatto, UMDNJ) was used as a nucleoplasmic marker. Where indicated, extracts were treated with 20U RNase A/ml extract (Invitrogen) by incubation at 37°C for 1 hour, just prior to immunoprecipitation.

### Immune Capture (IC) and Western Blot Experiments

500 µg of wild type procyclic nuclear extract was used for each IC sample. An affinity-purified antibody against the L5 peptide ^296^VAAVIERIRDRAK^308^ (Bethyl Laboratories) was used. Dynabeads (Invitrogen) were cross-linked to anti-L5 antibody using dimethyl pimelimidate (DMP, Thermo Scientific) following the manufacturer’s instructions. As a negative control, no antibody was added to one reaction to assess the degree of non-specific interactions with the Dynabeads. Nuclear extracts were added to the Dynabeads coated with antibody and incubated overnight at 4°C. Supernatants (S) were collected and the beads were washed five times with phosphate-buffered saline (PBS). The Dynabeads were then resuspended in SDS-PAGE sample buffer and boiled for five minutes to dissociate interacting proteins. Finally, samples were subjected to denaturing gel electrophoresis. To detect the presence of P34 and P37 in association with L5, the proteins were transferred to a nitrocellulose membrane. The membrane was blocked with 10% non-fat milk and incubated with an anti-P34/P37 affinity purified, polyclonal antibody previously described [10]. A secondary goat anti-rabbit antibody conjugated to horseradish peroxidase (HRP) was used for detection in conjunction with the SuperSignal West Pico Chemiluminiscent Substrate (Thermo Scientific). These experiments were repeated three times and representative results are shown.

For the IC with recombinant proteins, 100 ng of recombinant L5 were incubated with 100 ng of recombinant P34 or P37 in the absence or presence of stoichiometric amounts of *in vitro*-transcribed 5S rRNA and immunoprecipitated with Dynabeads cross-linked to the L5 peptide antibody as indicated above. The experiments were repeated a minimum of three times with different preparations of recombinant proteins and synthetic 5S rRNAs.

### Sequential Immune Captures

Immune capture experiments were performed as described above, except that following incubation with the nucleoplasmic extracts, the beads were washed with PBS and eluted using PBS with 1% SDS, 50 mM DTT and 10% β-mercaptoethanol as previously described [18]. After dilution and boiling for five minutes, samples were allowed to renature at room temperature. Then, these samples were subjected to a second round of IC as described above, with Dynabeads coated with the anti-P34/P37 antibody. Finally, reverse transcription was performed using 5S rRNA-specific primers and the products were resolved by agarose gel-electrophoresis and stained with ethidium bromide. These experiments were repeated three times and representative results are shown.

### Immunodepletion of Extracts

Protein A beads (50 µL slurry) were coated and cross-linked with 5 µg of anti-human L3 antibody (Abnova), previously shown to react specifically with *T. brucei* L3 [19]. Procyclic cell extracts (100 µg) were incubated with the beads at 4°C overnight with rotation. The supernatant was tested for L3 and tubulin by western blot analysis and analyzed using the immunoprecipitation protocol described above for an interaction between L5 and P34/P37.

### Recombinant Proteins

Both P34 (NRBD1, GenBank AF020695) and P37 (NRBD2, GenBank AF020696) were cloned into plasmid pQE-1 (QIAGEN) and expressed as histidine-fusion proteins in *E. coli* strain M15. Expression was induced with 1 mM IPTG for 5 hours at 37°C. Cell pellets were frozen and thawed, resuspended in lysis buffer (50 mM NaH_2_PO_4_, pH 8.0, 300 mM NaCl, 1% Triton X-100, 0.5 mM DTT, 10 mM imidazole) and incubated with lysozyme (1 mg/mL) for 30 minutes. Cells were lysed by sonication (six 10 second bursts at 75 W with a 10 second cooling period between bursts) and, if the lysate was viscous, DNase I was added to a final concentration of 5 µg/mL. After centrifugation at 10,000×g to remove cellular debris, the cell lysate was incubated with a 50% Ni-NTA agarose slurry (QIAGEN) at a 1∶4 slurry:lysate ratio for 1 hour at 4°C with gentle rotation. The mixture was packed in a column, and washed four times with wash buffer (same as lysis buffer with 20 mM imidazole). The recombinant protein was eluted with elution buffer (containing 250 mM imidazole) in four fractions of 500 µL each. Fractions containing recombinant protein (as visualized by SDS-PAGE with Coomassie blue staining) were pooled and desalted in a PD-10 column (GE) with storage buffer (10 mM Tris, pH 7.6, 150 mM KCl, 0.5 mM EDTA, 0.5 mM MgCl_2_, 1 mM DTT, 0.1 mM PMSF) and flash-frozen in 100 µL aliquots.

*T. brucei* ribosomal protein L5 (GenBank XM_822569) was cloned into pTrc-His1 (Invitrogen) for expression as a histidine-fusion protein. Our first attempts to produce active recombinant protein using the conditions described above were unsuccessful, in agreement with previous reports that L5 easily oxidizes and becomes inactive [20]. Therefore all solutions that came in contact with recombinant L5 were degassed and contained 1 mM DTT. Freeze/thaw cycles were avoided and proteins were used within two weeks while stored at 4°C.

Site-directed mutagenesis (Invitrogen) was used to mutate residue 291 (285 in yeast numbering) from alanine, encoded by GCA, to arginine, encoded by CGT. This protein is referred to as L5R in this study.

### Electrophoretic Mobility Shift Assay

5S rDNA (GenBank M14817.1) was amplified using primers 5ST3Fwd(5′ATTAACCCTCACTAAAGGGTACGACCATACTTGGCC 3′) and 5SRev (5′AGAGTACAACACCCCGGGT 3′) to generate a template that was used for T3 polymerase directed *in vitro* transcription (Maxiscript, Applied Biosciences) in the presence of [α-^32^P]UTP. The full-length, radiolabeled RNA was separated from truncated products and unincorporated nucleotides by denaturing gel electrophoresis and purified from the gel. Purified 5S rRNA was incubated with different concentrations of recombinant protein in binding buffer (10 mM Tris-HCl, pH 7.4, 150 mM KCl, 0.1 mM DTT, 0.1 mM EDTA, 0.1% NP-40) for 20 minutes at room temperature. After incubation, the reactions were applied onto a 6% native polyacrylamide gel (0.5× TBE, 5% glycerol) and electrophoresed at 100 V for 1.5 hours. Following electrophoresis, gels were dried and exposed to film and/or analyzed on a phosphorimager (BioRad). Quantification of the results was accomplished using GraphPad Prism 5. Experiments were repeated three times and representative results are shown.

### Filter Binding Assay

Filter binding assays with recombinant proteins were performed as described before [21], [22]. Briefly, a constant concentration of 5S rRNA (equivalent to 10,000 dpm, always lower than 0.5 nM) was used in all the reactions and increasing concentrations of recombinant protein were added in a total volume of 100 µL in binding buffer (10 mM Tris, pH 7.4, 1 mM EDTA, 100 mM NaCl, 0.1% NP40, 100 µg/mL BSA). After incubation for 20 minutes at room temperature, the reactions were applied onto pre-wetted nitrocellulose filters. The filters were washed twice with buffer, once with ethanol and then dried. A nylon filter underneath the nitrocellulose filters was used to capture unbound RNA. Radioactivity associated with the filters was measured with a phosphorimager. All reactions were performed in triplicate using two different preparations of recombinant protein. The bound fraction for each data point was tabulated as a ratio between the signal on the nitrocellulose filter and the total signal on both filters.

### RNA Capture Assays

*T. brucei* 5S rRNA was transcribed *in vitro* as described above in the presence of biotinylated UTP (at a 2∶3 ratio of labeled to unlabeled nucleotide). After gel purification using UV shadowing, the biotinylated rRNA was incubated with recombinant proteins L5 or L5R in binding buffer as described above. Dynabeads (Invitrogen) coated with streptavidin (or beads alone as controls) were used to capture the RNA and bound proteins. The precipitated fractions and the supernatants were analyzed by western blot as described above using the anti-L5 peptide antibody.

### RNA Co-immunoprecipitation Analysis

Studies were performed as previously described [11] using 500 µg of nuclear extracts and polyclonal sera directed against P34 and P37 or polyclonal sera directed against the yeast L5 protein (formerly L1) [23]. 1 µL of RNAseOUT (Invitrogen) was included in the precipitation reactions. Total RNA was isolated using TRIzol reagent (Invitrogen) and northern blot analysis was performed as before [24]. Densitometric analysis was performed as above. Assays were performed in triplicate.

## Results

### P34 and P37 Associate with L5 in Nuclear Extracts

**Figure 1:**
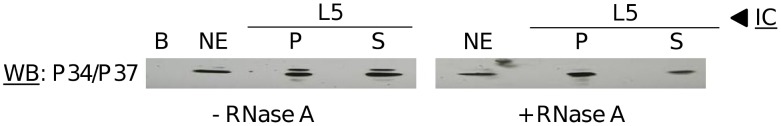
P34 and P37 interact with the L5 ribosomal protein in the nucleus in an RNase-resistant association. Nuclear extracts were immunoprecipitated with an anti-L5 antibody. The immunoprecipitate (P) and the supernatant (S) fractions were probed for the presence of P34 and P37 by western blot analysis. B: Beads alone (no antibody), NE: input nuclear extract. Samples on the right panel were treated with RNase A to disrupt interactions bridged by RNA.

Eukaryotic 5S rRNA is known to interact with only a select number of protein partners, the most conserved being the L5 ribosomal protein. L5 binds 5S rRNA in the nucleoplasm forming an extraribosomal particle that stabilizes 5S rRNA and directs it to the nucleolus [7]. Therefore, we wished to investigate whether the association of P34 and P37 with 5S rRNA occurs in the context of a complex with L5. We have previously reported the novel association between two trypanosome-specific factors, P34 and P37, and 5S rRNA ([11],[16]). It then becomes relevant to know if P34 and P37 also associate with L5. We first analyzed nuclear extracts to investigate whether an association could be detected in the cellular context in the presence of other factors. Immune capture experiments using an L5 peptide antibody were performed on nuclear extracts with or without addition of RNAse. Western analyses of the precipitate and the supernatant fractions were then performed using antibodies against P34/P37. As shown in Figure 1, (left panel), the beads alone (B) do not interact non-specifically with P34 and P37 (negative control) and the proteins are readily detected in the input fraction (NE, positive control). P34 and P37 can be detected in the supernatant fraction (S). Importantly, P34 and P37 can also be detected in the precipitate fraction (P lane). This indicates that part of the pool of P34 and P37 in procyclic cells is associated with the L5 ribosomal protein in the nucleus. Significantly, when RNase A is added to the extracts (Figure 1, right panel), P34 and P37 can still be found in the precipitate (P lane). This suggests that the interaction observed in nuclear extracts between L5 and P34 and P37 is not dependent on RNA. Although it appears as there is less P37 associated after RNase treatment, the signal observed is also lower in the extract lane.

### L5, P34 and P37 Associate in a Nucleoplasmic Particle with 5S rRNA

Most ribosomal proteins are found associated with rRNAs exclusively in the context of the ribosome. However, some extra ribosomal functions have been described for an increasing number of ribosomal proteins [25]. 5S rRNA is transcribed by RNA polymerase III in the nucleoplasm and it is necessary to stabilize it and transport it to the nucleolus for proper assembly into the nascent ribosome. This function is usually performed by the L5 protein. Therefore, we asked the question of whether the association between P34, P37, L5 and 5S rRNA, occurs in the ribosomal context or in a pre-ribosomal location which could suggest functional roles. Previous results from sucrose density analysis of nuclear extracts ([26] and Ciganda, unpublished results) showed that P34 and P37, 5S rRNA, and L5 were all present in a low molecular weight fraction. These results suggested that they participated in a small non-ribosomal complex.

**Figure 2:**
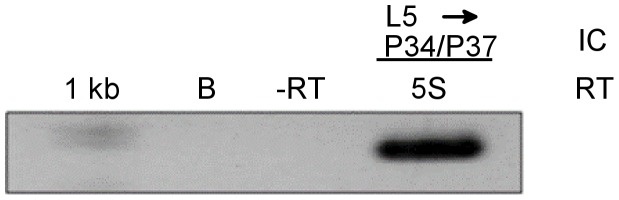
P34 and P37 form a preribosomal particle with 5S rRNA in the nucleoplasm. A sequential immune capture was performed on nucleoplasmic extracts with anti-L5 and anti-P34/P37 antibodies to enrich for complexes containing both proteins. RT was performed on these complexes to detect the presence of 5S rRNA. 1 kb: Molecular marker, B: Beads alone (no antibody), -RT: control without reverse transcriptase, NP: nucleoplasmic fraction.

We further investigated this question by performing biochemical fractionation of the nuclear material to obtain a highly purified nucleoplasmic fraction (determined by the absence of nucleolar markers, as in [19]). We assayed this purified nucleoplasmic fraction in a sequential immune capture experiment with L5 antibody followed by elution and precipitation with the P34/P37 antibody to enrich for complexes containing both L5 and P34 and/or P37. Finally, we performed reverse transcription with primers directed against 5S rRNA. These results are shown in Figure 2. When no specific antibodies are used, no signal for 5S rRNA is detected in the precipitate (lane B, beads alone). When no reverse transcriptase is used, no signal is detected either, indicating that there was no detection of nuclear DNA (lane –RT). Finally, precipitation with the specific L5 and P34/P37 antibodies followed by reverse transcription and PCR to amplify 5S rRNA demonstrate that there is a nucleoplasmic complex containing all three components (NP lane). Since L5 is known to aid in the stabilization and transport of 5S rRNA, the finding that P34 and P37 associate at such an early stage with the rRNA supports our hypothesis that P34 and P37 may be involved in this process.

**Figure 3:**
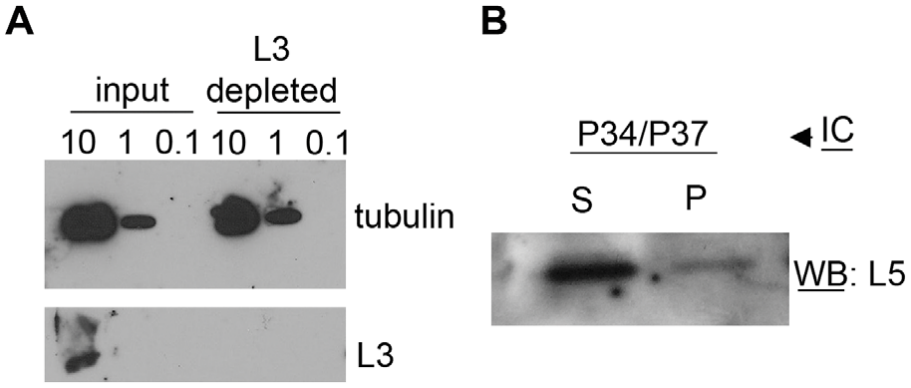
P34/P37 interact with L5 in extracts depleted of ribosomal proteins. Panel A: Western blot analysis of different dilutions (containing 10, 1 and 0.1 µg of total protein) of input and immunodepleted extracts with anti-tubulin (control) and anti-L3 antibodies. Panel B: Coimmunoprecipitation of immunodepleted extracts using anti-P34/P37 coated beads and anti-L5 antibody for the western blot analysis. S: Supernatant, P: precipitate.

Finally, we analyzed extracts which had been specifically depleted of ribosomal particles using L3-coated magnetic beads. The immunodepleted extracts were first tested to assess the degree and specificity of the depletion (Figure 3, Panel A). As expected, L3 is only detected in the input extract, but not in the depleted extract. Both the input extract and the depleted extract react with the anti-tubulin antibody at equivalent levels, showing that the depletion was specific. The depleted extract was analyzed by coimmunoprecipitation (Figure 3, Panel B) and an association between non-ribosomal P34/P37 and L5 was detected (P lane). This further supports the previous data showing that L5 interacts with P34/P37 independently of other ribosomal factors.

### P34 and P37 Interact with L5 in a Direct Association, which can be Modulated by the Presence of 5S rRNA

**Figure 4:**
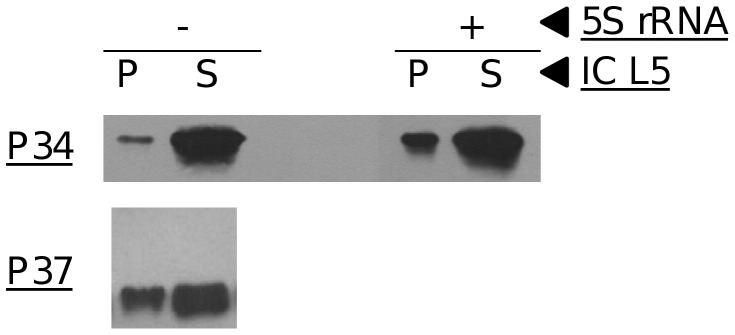
P34 and P37 interact with L5 directly in a 5S rRNA-modulated association. Recombinant proteins P34 or P37 were incubated with L5 in the absence (left) of *in vitro-*transcribed 5S rRNA and then precipitated with an anti-L5 antibody. Immunoprecipitate (P) and supernatant (S) fractions were then analyzed by western blot using an antibody against P34/P37. Recombinant protein P34 was incubated with L5 in the presence of *in vitro* transcribed 5S rRNA (right) and then precipitated as above.

We then proceeded to isolate the interaction by investigating the behavior of recombinant proteins *in vitro* in the absence of other cellular factors. In these experiments, we utilized purified recombinant L5, P34 and P37 proteins. Immune capture fractions (Figure 4, left) using a peptide antibody against L5 were subsequently analyzed using the P34/P37 antibody. P34 and P37 are found in the supernatant (S), and, significantly, they are also found in the precipitate for each individual incubation (P). The results indicate that there is a direct interaction between P34 and P37 with L5 and that no additional *T. brucei* factors, protein or RNA, are needed to establish or maintain this association. These results correlate with the previous immune capture experiment where P34 and P37 retained their association with L5 after RNase treatment (Figure 1).

However, these *in vivo* and *in vitro* experiments did not exclude the possibility that 5S rRNA may be important for efficient binding between P34, P37 and L5. To further clarify this point, we performed the *in vitro* immune capture experiments with L5 and P34 in the presence or absence of *in vitro* transcribed 5S rRNA. Addition of 5S rRNA to the *in vitro* reaction increases the levels of P34 in the immune capture precipitate (Figure 4, right). This increase in binding was not seen consistently with P37, but the significance of this observation is not known. These results suggest that 5S rRNA may augment the bridging and stabilization of the interaction between L5 and P34.

### The Sequence of *T. brucei* L5 Differs from the Consensus at Key Positions

**Figure 5:**
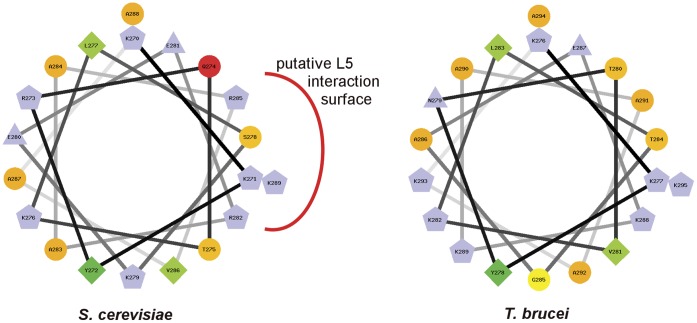
The C-terminal domain of L5. The C-terminus of the yeast L5 protein is displayed and compared to the same region of the *T. brucei* L5 protein. The arc around the alpha helix indicates the potential site of a 5S rRNA interaction. Basic residues are marked in blue.

**Figure 6:**
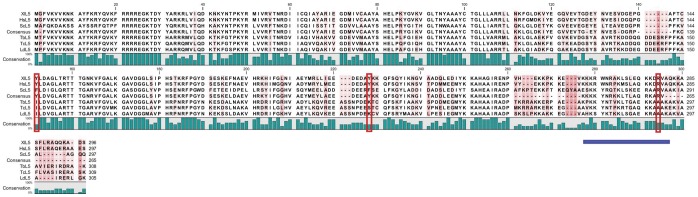
L5 in trypanosomatids differs from the eukaryotic consensus at potentially key positions. Multiple alignment of the C-terminal domains of L5 proteins across phylogeny. 447 L5 sequences were retrieved from the NCBI protein database limiting results to eukaryotic, non-mitochondrial, non-putative sequences, and then aligned using Clustal. The resulting consensus sequence is aligned here with the L5 proteins discussed in this work.Xl: *Xenopus laevis* (NP_001079437.1), Hs: *Homo sapiens* (NP_000960.2), Sc: *Saccharomyces cerevisiae* (P26321.4), Tb: *T. brucei* (Tb09.244.2740), Tc: *T. cruzi* (EAN92842), Ld: *Leishmania donovani* (CBZ38090.1). The red boxes highlight substitutions in trypanosomatids. The blue bar indicates the position of the alpha helix shown in Figure 5.

The unexpected presence of two trypanosome-specific factors in a highly conserved RNA:protein complex led us to question how such an arrangement would be advantageous or even necessary for trypanosomes. Our previous results show that in the absence of P34 and P37, procyclic *T.brucei* cells exhibit phenotypic characteristics that indicate that the biogenesis, transport and/or stability of 5S rRNA have been compromised [27]. In *Saccharomyces cerevisiae,* the absence of L5 has been linked to very similar phenotypic consequences [9]. Therefore, we analyzed the structural elements in L5 that have been implicated in 5S rRNA binding, and found that, although overall there is good conservation between TbL5 and the eukaryotic consensus (54% identity with the *Xenopus laevis* protein), some of the substitutions are located in regions previously implicated in binding to 5S rRNA. As shown in Figure 5, the C-terminus of yeast L5 (left) has been proposed to contain an alpha helix with several positively charged amino acids positioned on the same side of the helix, creating a pocket for 5S rRNA interaction [28]. For comparison, the same side of the helix in *T. brucei* (Figure 5, right) contains a less dense concentration of positive charge. These basic amino acids found in yeast are well conserved in other eukaryotes (Figure 6, the helix is indicated by a solid blue line). However, in trypanosomatids a highly conserved arginine within this helix has been substituted with a non-charged alanine (yeast position 285, Figure 6). In addition, an aromatic residue at yeast position 145 which contributes modestly to binding [29] is substituted in trypanosomes for a non-aromatic residue. This suggests that *T. brucei* L5 may not bind 5S rRNA as efficiently as the yeast homologue, thus supporting the need for additional protein co-factors such as P34 and P37.

### TbL5 Binds 5S rRNA with a Lower Kd than that of Other Eukaryotic L5 Proteins

**Figure 7:**
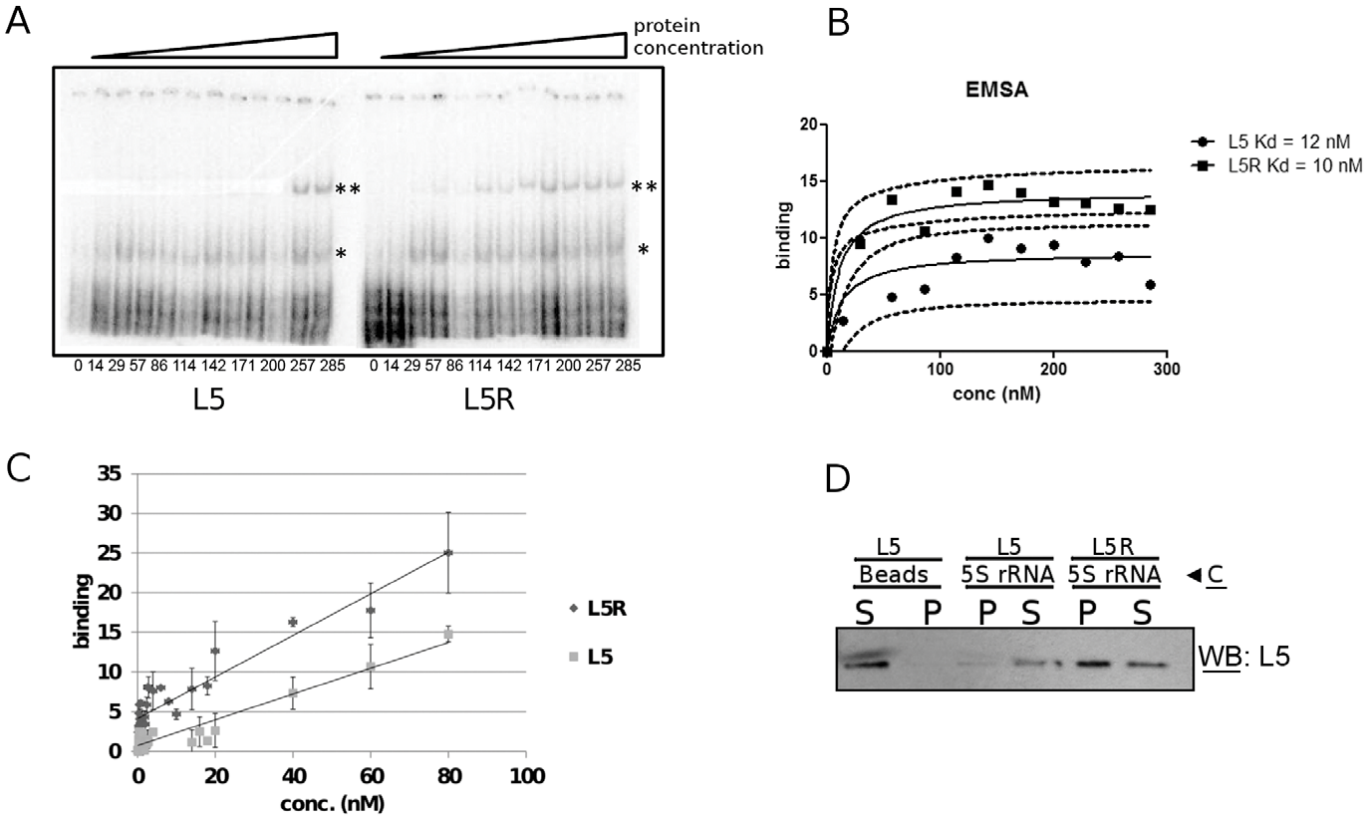
A mutation in the C-terminal domain of *T. brucei* L5 restoring a consensus arginine increases affinity for 5S rRNA. Panel A: EMSA using radiolabeled 5S rRNA and recombinant *T. brucei* wild-type (left) and arginine mutant (right) proteins. The asterisk indicates the monomer:RNA complex and the double asterisk indicates the dimer:RNA complex. The numbers below indicate concentration of protein in nM. Panel B: Quantification of the data in Panel A. The dashed lines indicate 90% confidence intervals for the fitted curves. Panel C: Filter binding assay of reactions with recombinant L5 or L5R and radiolabeled 5S rRNA. The range of concentrations was chosen to represent binding to monomer L5. Panel D: RNA capture of recombinant L5 and L5R bound to biotinylated 5S rRNA. C: Capture, P: precipitate, S: supernatant.

To investigate if these substitutions have any effect in the binding parameters of TbL5 to 5S rRNA, we performed EMSAs with recombinant L5 from *T. brucei*. Figure 7, Panel A, left shows that, as the concentration of recombinant protein is increased, formation of a complex is observed (indicated by the single asterisk). The complex is first detectable at concentrations of 4 nM for the *T. brucei* L5 protein (lane 6, left panel). Quantification of the bound fraction is shown in Figure 7, Panel B (circles). The Kd value calculated from these experiments is 12 nM, higher than the 2 nM value previously reported for the well characterized *X. laevis* L5 ([6], [20], [29], [30] and Ciganda, unpublished data) We conclude that, compared to the *Xenopus* protein, TbL5 has an increased dissociation constant value for 5S rRNA, implying a lower affinity with the difference being half an order of magnitude.

### *T. brucei* L5 Binds a Relatively Low Fraction of the Nuclear 5S rRNA

**Figure 8:**
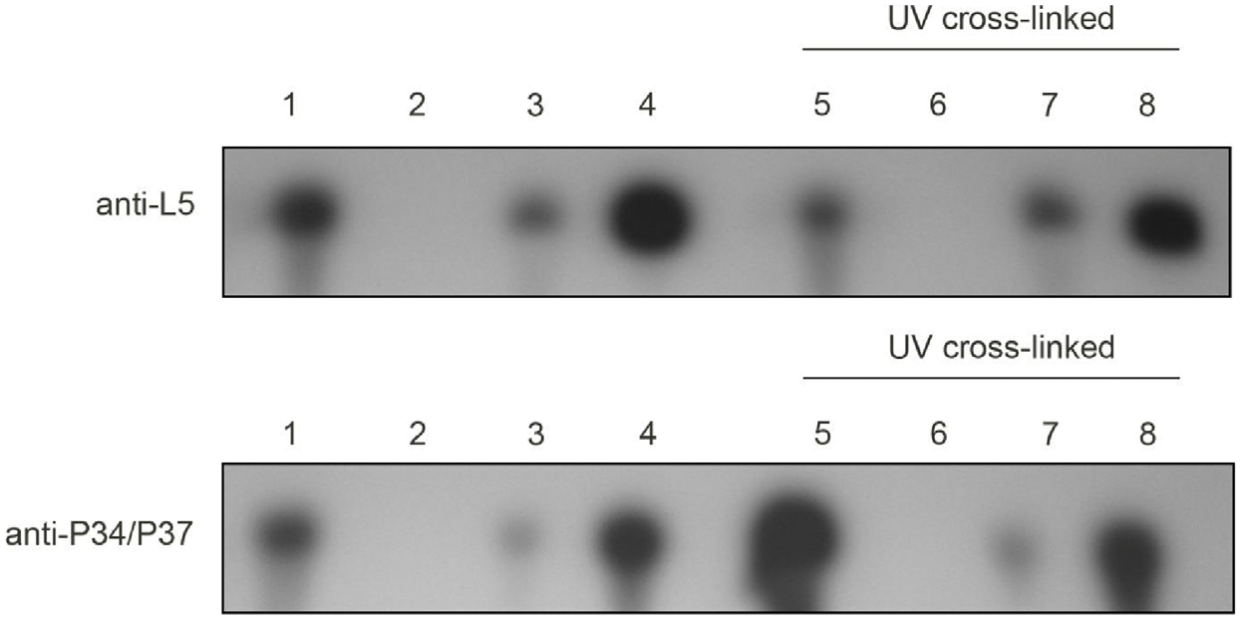
*T. brucei* L5 binds a low percentage of the total nuclear 5S rRNA. Northern blot analysis of 5S rRNA within immunoprecipitate samples utilizing either anti-L5 or anti-P34/P37 antibodies to immuno-precipitate these proteins and associated 5S rRNA from wild type nuclear extracts. Lane 1: total RNA, lane 2: pre-immune sera control, lane 3: experimental lane, lane 4: supernatant fraction. Lanes 5–8: same as lanes 1–4, respectively, but UV cross-linking was performed prior to immunoprecipitation.

In order to further explore the significance of these sequence substitutions, we analyzed binding of the native L5 protein in procyclic cells. Co-immunoprecipitation of nuclear extracts was performed with anti-sera to either the P34/P37 proteins or the L5 protein. We found that 23.91±.0.2% of 5S rRNA co-precipitates with *T. brucei* L5 (Figure 8, top panel, lane 3). Further stabilization of the complexes by UV cross-linking of the nuclear extracts prior to experimentation did not result in significant alterations of the immunoprecipitated material (lane 7). This is in sharp contrast to the situation in mammalian cells, where 95% of 5S rRNA is bound by the L5 ribosomal protein [7]. Immunoprecipitation with the anti-P34/P37 antiserum (Figure 8, bottom panel) shows that 26.08%±0.2% of the 5S rRNA within the same nuclear extracts interacts with P34 and P37 (lane 3). Our previous sequential capture experiments indicate that this pool of 5S rRNA bound to P34 and/or P37 must overlap at least partially with the pool of 5S rRNA bound to L5.

### Substituting an Alanine Residue in the C-terminus to a Consensus Arginine Increases Binding Affinity of TbL5 for 5S rRNA

We next wished to explore the possibility that the amino acid variations detected before might be involved in these differences in the *in vitro* affinity of L5 for 5S rRNA and in the *in vivo* abundance of the RNP.

We reasoned that if the substitutions we observed are responsible for the lower binding affinity of TbL5 for 5S rRNA, then restoring one or more of these amino acids to the eukaryotic consensus sequence would be associated with an increase in binding affinity for 5S rRNA. To test this prediction of our hypothesis, we generated a mutant of TbL5 in which the codon for alanine residue at position 285 (yeast numbering) has been replaced with a codon for a basic arginine as in the yeast C-terminus. We tested this recombinant protein in EMSAs alongside the wild type TbL5 for binding of 5S rRNA (Figure 7). As we increase the concentration of protein in the reaction, a complex is observed (Panel A, single asterisk). Both wild type L5 and the arginine substitution mutant protein behave similarly in the formation of this complex. However, a second complex results from dimerization at higher concentrations (Panel A, double asterisk). Because the presence of the dimer at concentrations above 100 nM made quantification difficult, we focused on the initial portion of the binding curve for analysis in filter binding assays (Figure 7, Panel C). At these concentrations, the rRNA is bound by the monomer form and the dimer has not yet formed. The fraction of 5S rRNA associated with the L5R protein at all data points was higher than the fraction associated with the wild type L5 protein by up to 50%. Finally, the association was further analyzed by co-precipitation experiments using streptavidin coated beads, biotinylated 5S rRNA and recombinant L5 or L5R proteins. As shown in Panel D, beads alone used as a negative control are not able to capture L5 in the reaction precipitate (P). When streptavidin-coated beads are used to capture biotinylated 5S rRNA and all bound protein, L5R is co-precipitated more efficiently than wild-type L5. Thus, the single amino acid difference between *T. brucei* L5 and the eukaryotic consensus accounts, at least in part, for the decreased affinity of *T. brucei* L5 for 5S rRNA.

## Discussion

The presence of essential, trypanosome-specific factors in association with 5S rRNA led us to speculate that further associations with the L5 ribosomal protein might play a role in the function of P34 and P37. Because the relevant context for the early stages of ribosome assembly is the nucleus, we first utilized nuclear extracts and we demonstrated that P34 and P37 associate with the L5 ribosomal protein (Figure 1). This association could be direct, or it could be mediated by other factors, protein or RNA. Treatment of the nuclear extracts with RNase did not have an effect on the interaction between P34 and P37 and L5. This indicates that either RNA is not required for the formation of the complex or that, upon formation of the complex the RNA component becomes dispensable (or inaccessible to RNase A).

In eukaryotic cells, L5 shuttles to the nucleus where it binds 5S rRNA shortly after its transcription in the nucleoplasm, and then localizes to the nucleolus in the context of an RNP. P34 and P37 are predominantly nuclear proteins [31] and we have recently reported their association with specific ribosomal components at defined steps in ribosome biogenesis in the nucleolus [19]. In addition, we have determined that P34 and P37 remain associated with cytoplasmic ribosomes in wild type *T. brucei* procyclic cells [27]. Therefore, there are several opportunities for an interaction between L5 and P34 and P37 to occur. Interactions within each subcellular compartment have specific functional implications. For example, if the interaction only occurs within the nucleolus, a role in the incorporation onto the nascent ribosomal particle is immediately suggested.

In previous experiments we found that P34/P37 co-sediment with L5 and 5S rRNA in both high and low molecular weight complexes ([27] and Ciganda, unpublished results). The presence of the low molecular complexes suggested the presence of these components within a non-ribosomal complex. Through biochemical fractionation and immune capture experiments, we demonstrated that the association between P34, P37, L5 and 5S rRNA occurs in the nucleoplasm (Figure 2). These complexes are not part of the mature ribosome, as indicated by immunodepletion experiments (Figure 3). This strongly supports a role in the early stages of ribosome biogenesis. In conjunction with our previous data that cells depleted of P34 and P37 contain destabilized 5S rRNA, a model begins to emerge in which P34 and P37 are necessary factors in the early binding and stabilization of 5S rRNA.

To investigate whether additional accessory factors are necessary to form this nucleoplasmic RNP, we analyzed the interaction *in vitro* with recombinant proteins. Under these conditions, we were able to show (Figure 4) that no additional factors are required to establish or maintain the association between P34, P37 and L5. Interestingly, addition of 5S rRNA (Figure 4) to the complex enhanced the protein-protein associations at least for the L5:P34 protein complex, suggesting conformational changes upon RNA binding, and strengthening the concept of a stable, trimolecular RNP. Results from other eukaryotes have shown that the conformation of L5 is dependent on the presence of 5S rRNA [30].

Even though a complete description of the interaction between L5 and 5S rRNA is still lacking, several studies have examined the role of specific domains and amino acids in the binding of 5S rRNA. Some researchers have postulated a role for the N-terminal domain of L5 in the binding of 5S rRNA. In these models, 5S rRNA binds L5 as it is being translated (N-terminus first) in polysomes in the cytoplasm, where it plays a chaperone-like role [32]. A study using deletion mutants also mapped the 5S rRNA-binding domains to the N-terminus of L5 [33].

However, others have found very little binding of 5S rRNA in the absence of the C-terminal domain of L5 in both rat and *Xenopus* [34]. In yeast, a peptide derived from the C-terminus of L5 was shown to retain 5S rRNA binding activity [35]. Further studies focused on a putative alpha helix with a high density of basic amino acids arranged on one side of the helix within this C-terminal peptide [36]. Mutations in these basic amino acids reduced or obliterated 5S rRNA stability and cell viability. Deletions within the L5 C-terminal domain also interfere with assembly of functional ribosomes [23].

Interestingly, we observed that one of these conserved basic amino acids within the C-terminal domain of L5 is substituted for alanine in the genome of the trypanosomatids (Figures 5 and 6). Other substitutions, which could have functional implications, are also found in trypanosomes. In particular, aromatic amino acids involved in hydrophobic interactions with RNA bases [29], [30] are not well conserved.

To analyze whether this observation has any experimental consequences, we studied 5S rRNA binding of the *T. brucei* recombinant L5 protein (Figure 7). The *T. brucei* protein binds 5S rRNA with a Kd of 12 nM, half an order of magnitude above the published Kd values of the *X. laevis* protein of 2 nM, indicating a lower affinity ([6] and Ciganda, unpublished results). We have also demonstrated that the fraction of 5S rRNA bound to L5 is unusually low in *T. brucei.* Only about 24% of the total 5S rRNA was found to be in association with *T. brucei* L5 (Figure 8), when 95% of the 5S rRNA is found in association with mammalian L5 [7]. Early work performed on a 7S RNP in *T. brucei* [37] had noted that it contains a relatively low amount of 5S rRNA and that some of its biochemical characteristics are different from those of the mammalian 7S RNP (resistance to high salt being the most salient). Strikingly, in previous experiments with cells lacking P34 and P37 (where 5S rRNA has been depleted), we did not detect changes in the expression of L5 [38]. This suggests that the feedback loop that normally couples 5S rRNA synthesis with L5 expression [39] is not functional in *T. brucei.* In addition, our results indicate that L5 by itself is not able to promote 5S rRNA stability in the context of depletion of P34 and P37.

Finally, we were able to show that mutating the alanine at the C-terminus to an arginine has the effect of increasing binding affinity for 5S rRNA (Figure 7). It has been reported that the human L5 protein can form homodimers [40] and our results suggest that the main difference brought about by this mutation would be an increased affinity for 5S rRNA in the homodimer state of *T. brucei* L5 (Figure 7, Panel A). An estimation of the increase in binding affinity of the total mutant protein (dimer and monomer) versus wild-type protein (dimer and monomer) shows that the single substitution mutant achieves a 16% increase in affinity when both dimers and monomers are quantified (Panel B). If only the binding of monomers at low concentrations is analyzed, the relative increase in the binding of the substitution mutant is between 25 and 50% higher (Panel C). We believe other amino acid substitutions, particularly aromatic amino acids, may still be responsible for the remaining differences in 5S rRNA binding between the arginine substitution mutant L5R and wild-type *X. laevis* L5. In addition, biotinylated 5S rRNA is coprecipitated with L5R more efficiently than with wild type *T. brucei* L5 (Panel D).

We speculate that the lower affinity of the *T. brucei* L5 protein makes it necessary for the trypanosomatids to compensate by recruiting P34 and P37 to the role of binding, stabilizing and trafficking 5S rRNA to the nucleolus. This hypothesis also explains our observation that depletion of P34 and P37 levels in *T. brucei* leads to an inviable phenotype caused by 5S rRNA instability and ribosomal biogenesis defects, similar to that observed in yeast, when L5 is knocked out [9]. After trafficking to the nucleolus, P34 and P37 remain associated with the nascent 60S subunit, and are essential for its export to the cytoplasm [19].

It is possible that the presence of P34/P37-type proteins in the trypanosomatids precedes the divergence of the L5 C-terminal domain. P34 and P37 may have evolved an affinity for 5S rRNA together with their role in the 60S subunit export [19]. This association may have allowed the L5 C-terminal domain to better tolerate mutations later in evolution.

Further experiments in our laboratory are under way to define the relative importance of the L5 substitution mutants in an *in vivo* context. In particular, we would like to know if a more consensus-like L5R protein is able to complement the defects in ribosomal biogenesis observed in our P34/P37 RNAi cell line. Furthermore, even though the L5 protein is known to be essential in other eukaryotes [23], the presence of P34 and P37 could make trypanosomes at least partially refractory to a depletion of their L5 levels. This intriguing possibility is also currently being addressed through RNAi studies of L5 in trypanosomes.
